# Magnitude Pruning of Large Pretrained Transformer Models with a Mixture Gaussian Prior

**DOI:** 10.6339/24-jds1156

**Published:** 2024-11-26

**Authors:** Mingxuan Zhang, Yan Sun, Faming Liang

**Affiliations:** 1 Department of Statistics, Purdue University, West Lafayette, IN 47907, USA; 2 Department of Biostatistics, Epidemiology, and Informatics, University of Pennsylvania, Pennsylvania, PA 19104, USA

**Keywords:** consistency, large language model, sparsity, stochastic transformer, transformer

## Abstract

Large pretrained transformer models have revolutionized modern AI applications with their state-of-the-art performance in natural language processing (NLP). However, their substantial parameter count poses challenges for real-world deployment. To address this, researchers often reduce model size by pruning parameters based on their magnitude or sensitivity. Previous research has demonstrated the limitations of magnitude pruning, especially in the context of transfer learning for modern NLP tasks. In this paper, we introduce a new magnitude-based pruning algorithm called mixture Gaussian prior pruning (MGPP), which employs a mixture Gaussian prior for regularization. MGPP prunes non-expressive weights under the guidance of the mixture Gaussian prior, aiming to retain the model’s expressive capability. Extensive evaluations across various NLP tasks, including natural language understanding, question answering, and natural language generation, demonstrate the superiority of MGPP over existing pruning methods, particularly in high sparsity settings. Additionally, we provide a theoretical justification for the consistency of the sparse transformer, shedding light on the effectiveness of the proposed pruning method.

## 1 Introduction

Large pretrained transformer models have emerged as powerful tools for a variety of downstream natural language processing tasks, from natural language generation to question answering (Radford et al., 2019; Brown et al., 2020). These pretrained models have grown exponentially in size, often comprising hundreds of millions, or even billions, of parameters (Devlin et al., 2019; He et al., 2021; Lewis et al., 2019; Touvron et al., 2023). While their capabilities are undeniably impressive, the computational and storage requirements for such large models are becoming increasingly prohibitive (Strubell et al., 2020).

Score-based pruning, a technique that involves removal of non-expressive parameters based on their importance score rankings, presents a promising avenue for model compression. It has the potential to significantly reduce model size with minimal impact on performance.

Based on the definition of pruning scores, the pruning methods can be classified into distinct categories, such as magnitude-based (zeroth-order) pruning methods (Han et al., 2015a,b; Zhu and Gupta, 2017; Louizos et al., 2017; Wang et al., 2020) and sensitivity-based (higher-order) pruning methods (Molchanov et al., 2019; Ding et al., 2019; Sanh et al., 2020; Liang et al., 2021; Zhang et al., 2022; Kurtic et al., 2022; Li et al., 2023). On the other hand, if the classification is made based on the strategies employed, the methods fall into categories such as one-shot pruning (Lee et al., 2018; Frankle and Carbin, 2018; Chen et al., 2020; Liang et al., 2021; Zafrir et al., 2021) and iterative pruning (Han et al., 2015a; Zhu and Gupta, 2017; Louizos et al., 2017; Sanh et al., 2020; Zhang et al., 2022; Li et al., 2023).

It has long been argued and experimentally demonstrated that magnitude-based pruning methods struggle to retain expressive parameters, particularly in high-sparsity settings. Furthermore, when it comes to transfer learning with large pretrained models, which are now the benchmark for state-of-the-art downstream NLP tasks, their effectiveness is reduced. As a result, models pruned using magnitude-based methods often exhibit diminished generalization performance (Sanh et al., 2020).

Recently, Bayesian sparse deep learning has made significant progress through a series of works (Liang et al., 2018b; Sun et al., 2022b,a, 2021; Zhang et al., 2023), demonstrating its potential in deep learning for both statistical inference and model sparsification. By adopting the mixture Gaussian prior (MGP) for the parameters of the neural network, they developed a magnitude-based one-shot pruning method and achieved state-of-the-art performance in pruning both convolutional neural networks (Sun et al., 2022a, 2021) and recurrent neural networks (Zhang et al., 2023). These early experimental results on small-scale models have once again sparked hope for magnitude-based pruning methods. However, their methods have not yet been evaluated on large transformer models across different tasks and datasets. Moreover, as we will discuss in Section 2.3, several key challenges prevent us from directly adopting their methods for pruning larger models.

In this work, we introduce MGPP, a magnitude-based iterative pruning algorithm that is both simple and effective. To validate its performance, we conducted extensive experiments across three key downstream tasks: natural language understanding, question answering, and natural language generation. Our evaluations span three types of pretrained transformer-based language models, DeBERTaV3_base_ (He et al., 2021), BERT_base_ (Devlin et al., 2019), and BART_large_ (Lewis et al., 2019). Our results indicate that when guided by an appropriate prior, magnitude-based methods can outperform existing state-of-the-art pruning methods. Additionally, we provide a loose justification for the consistency of the sparse transformer, shedding light on its effectiveness.

The remaining part of the paper is organized as follows. Section 2 provides preliminary descriptions for related concepts and methods in the literature. Section 3 describes the proposed method and justifies its validity. Section 4 reports numerical experiments. Section 5 concludes the paper with a brief discussion.

## 2 Preliminaries

### 2.1 Pruning Scores

An essential component of effective pruning is accurately identifying non-expressive parameters through their importance score rankings. Consider a model defined by a set of parameters $\theta ={\left(\theta_{1},\ldots ,\theta_{d}\right)}^{T}\in R^{d}$, each associated with an importance score. Let $S={\left(S_{1},\ldots ,S_{d}\right)}^{T}\in R^{d}$ denote the corresponding score vector. Score-based pruning methods eliminate parameters based on these scores, with parameters assigned lower scores being prioritized for removal. As outlined in the Introduction, score-based pruning methods fall into two primary categories, namely, magnitude-based methods and sensitivity-based methods.

#### Magnitude-Based (Zeroth-Order) Methods

(Zhu and Gupta, 2017; Wang et al., 2020; Chen et al., 2020; Zafrir et al., 2021), which determine the parameters to prune based on their magnitudes. For a given parameter $\theta_{j}$, the score is defined as $S_{j}=|\theta_{j}|$. Among these methods, gradual magnitude pruning (GMP), introduced by Zhu and Gupta (2017), is particularly notable for its effectiveness and simplicity. This widely adopted pruning baseline has inspired the development of numerous subsequent methods, see e.g., Chen et al. (2020) and Zafrir et al. (2021).

#### Sensitivity-Based Methods

(Sanh et al., 2020; Zhang et al., 2022; Kurtic et al., 2022; Li et al., 2023), which incorporate higher-order information, such as gradients and Hessian, to assess the impact of pruning on the loss function ℒ. The first-order pruning methods utilize gradient-based information. Notable examples include movement pruning (MvP) (Sanh et al., 2020) and PLATON (Zhang et al., 2022). The former removes model parameters that are moving towards zero, and the latter is designed to capture the uncertainty of model parameters’ importance scores during the pruning process. In downstream pruning scenarios, particularly for BERT-like language models, PLATON is recognized as state-of-the-art, significantly outperforming other baselines, including MvP. The second-order pruning methods (LeCun et al., 1989; Singh and Alistarh, 2020; Frantar et al., 2021; Kurtic et al., 2022) utilize Hessian-based information. To circumvent the costly approximation of the inverse Hessian, Singh and Alistarh (2020) introduced the WoodFisher method, and Frantar et al. (2021) introduced the M-FAC method. However, Kurtic et al. (2022) showed that the WoodFisher method is computationally infeasible at the scale of BERT, and while the M-FAC method scales effectively, it yields inferior pruning results. In response, they proposed a general second-order pruning method, Optimal BERT Surgeon (oBERT), which achieves state-of-the-art performance in upstream pruning scenarios.

While the zeroth-order methods are simple, scalable, and often serve as standard baselines, they are consistently outperformed by higher-order methods, particularly in downstream pruning scenarios and at high sparsity levels. However, as discussed above, the performance gains from utilizing higher-order information come at the cost of additional memory and computational resources. For a model with d parameters, PLATON requires an extra O(3d) memory to maintain three additional states: the average importance scores between consecutive pruning operations, and the exponential average of both the importance scores and the corresponding upper confidence bound. For the BERT_BASE_ model, with d=85 million parameters, managing these states is feasible. However, scaling to larger models, such as Llama-7b/70b (Touvron et al., 2023), necessitates approximately an additional 84GB/840GB of GPU memory.

The memory requirement for oBERT is O(Bd), where B is a hyperparameter representing the width of the diagonal block-wise approximation of the empirical Fisher matrix. For the BERT_BASE_ model, setting B=50 results in an additional memory requirement of about 17GB. This demand is manageable for BERT_BASE_ but becomes unscalable for larger models. The runtime complexity of oBERT is O(mBd), where m denotes the number of gradient outer products used to approximate the Hessian; for the BERT_BASE_ model, m is set to 1024.

### 2.2 Pruning Strategies

Pruning strategies can be classified into one-shot pruning and iterative pruning. In one-shot pruning, the sparsity pattern is predetermined using the scores of a fully-trained, dense model. A sparse model is then trained with pruned parameters fixed, a technique often termed “rewinding.” However, choosing which parameters to prune based on a fully-trained model overlooks the complex dynamics of training. As a result, parameters that are expressive may be unfairly eliminated at the early stage of training.

On the other hand, iterative pruning jointly performs training and pruning. The sparsity pattern is dynamically updated, offering the model an opportunity to recover from previous pruning decisions. Additionally, the sparsity level can be gradually increased during training through sparsity schedulers, such as the cubic sparsity scheduler (Zhu and Gupta, 2017; Sanh et al., 2020; Zafrir et al., 2021; Zhang et al., 2022; Kurtic et al., 2022; Li et al., 2023) given as follows:

(1) v(t)={0t<ti,v(T)−v(T)(1−t−titf−ti)3ti≤t≤tf,v(T)tf<t≤T,}

where $v^{\left(t\right)}$ is the sparsity level at the t-th training step, increasing from an initial value 0 to a final level $v^{\left(T\right)}$ over a period of $T-t_{i}-t_{f}$ steps following a warm-up of $t_{i}$ steps. In practice, instead of performing pruning at every training step before reaching the target sparsity level, one can also choose to prune every Δt steps (Zhang et al., 2022; Li et al., 2023).

### 2.3 Mixture Gaussian Priors in Bayesian Sparse Deep Learning

The mixture Gaussian prior (MGP) has recently attracted significant attention in the field of Bayesian sparse deep learning (Sun et al., 2022a). Formally, it models each parameter of the network using a mixture Gaussian prior, defined as follows:

(2) $$\theta_{j}∼\lambda \cdot 𝒩(0,\sigma_{1}^{2})+(1-\lambda )\cdot 𝒩(0,\sigma_{0}^{2}),$$

where λ∈(0,1) is the mixture proportion, $\sigma_{0}^{2}$ is typically set to a very small value, whereas $\sigma_{1}^{2}$ is usually assigned a relatively larger value. In what follows, we denote the prior density function of $\theta_{j}$ as $\pi (\theta_{j};\lambda ,\sigma_{0}^{2},\sigma_{1}^{2})$. Furthermore, we assume that all model parameters are *a priori* independent, i.e., $\pi (\theta ;\lambda ,\sigma_{0}^{2},\sigma_{1}^{2})=\prod_{j=1}^{d}\pi (\theta_{j};\lambda ,\sigma_{0}^{2},\sigma_{1}^{2})$.

This particular prior has been shown to offer several theoretical advantages under the Bayesian framework. These include posterior consistency, structure selection consistency, and asymptotic normality of predictions for both i.i.d. data (Sun et al., 2022a, 2021) and timeseries data (Zhang et al., 2023). These properties make it useful in various applications like variable/model selection, uncertainty quantification, and model sparsification.

Next, we will discuss how this prior is used to prune models. Essentially, the prior serves as a form of regularization, imposing penalty on model parameters. During training, the learning objective becomes

(3) $$ℒ(D_{n},\theta )-\frac{1}{n}\;\log(\pi (\theta ;\lambda ,\sigma_{0}^{2},\sigma_{1}^{2})),$$

where n denotes the size of the training dataset $D_{n}$, and $ℒ(D_{n},\theta )$ represents the negative log-likelihood function of the deep neural network. To facilitate the application of gradient-based optimization algorithms for minimizing (3), we provide the following numerically stable expression of the gradient of log-prior, despite its straightforward derivation:

(4) $$\nabla_{\theta_{j}}\;\log(\pi (\theta_{j};\lambda ,\sigma_{0}^{2},\sigma_{1}^{2}))=-\left(\frac{\theta_{j}}{\sigma_{0}^{2}}g(\theta_{j})+\frac{\theta_{j}}{\sigma_{1}^{2}}[1-g(\theta_{j})]\right),$$

where

$$g(\theta_{j})={\left(\text{exp}\{c_{2}\theta_{j}^{2}+c_{1}\}+1\right)}^{-1},$$

with $c_{1}=\text{ln}(\lambda )-\text{ln}(1-\lambda )+0.5\;\text{ln}(\sigma_{0}^{2})-0.5\;\text{ln}(\sigma_{1}^{2})$ and $c_{2}=0.5∕\sigma_{0}^{2}-0.5∕\sigma_{1}^{2}$.

**Figure 1:**
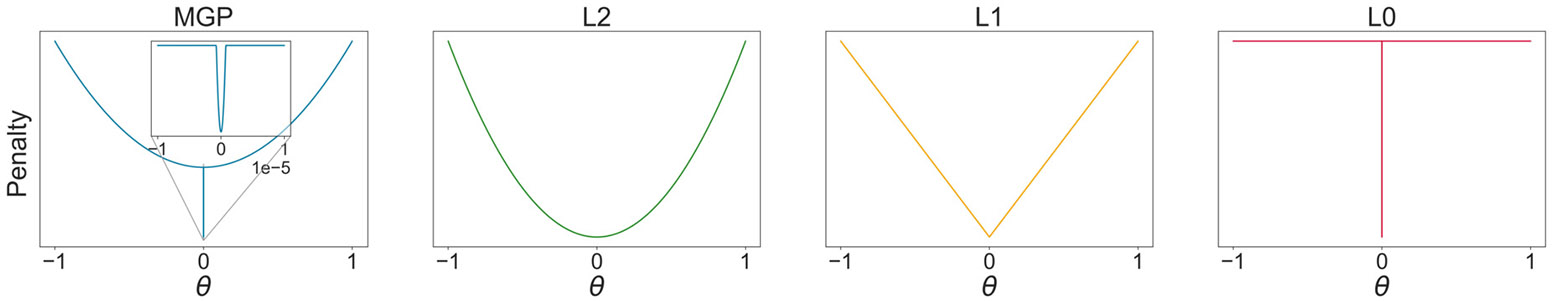
Visualization of the penalty functions across various regularization methods: the MGP displayed in the plot corresponds to $-\text{log}(\pi (\theta ;\lambda =1\times 10^{-6},\sigma_{0}^{2}=1\times 10^{-7},\sigma_{1}^{2}=0.1))$, where a zoomed-in view for the region near zero is provided. Unlike $L_{0}$ regularization, which is not differentiable and requires the use of gradient estimators (Louizos et al., 2017), the MGP is differentiable across the entire parameter space.

The guidance from the MGP is conveyed through the gradients. The degree of penalty, which serves as the force pushing the model parameters toward zero, can be quantified by the absolute value of the gradient. A comparison between $L_{0}$, $L_{1}$, $L_{2}$, and MGP is presented in Figure 1.

The MGP acts as a piece-wise $L_{2}$ regularization, imposing different penalties across various regions of the parameter space. On a larger scale, the MGP applies penalties to parameters in a manner similar to $L_{2}$ regularization. In contrast, near zero in the small-scale region, the MGP imposes a more substantial penalty, setting it apart from $L_{2}$ regularization. In Section S1 of the Supplement, we illustrate and visualize how λ, $\sigma_{0}^{2}$, and $\sigma_{1}^{2}$ affect the landscape of the MGP.

According to the definitions provided in Sections 2.1 and 2.2, previous methods employing MGP (Sun et al., 2022a, 2021, 2022b; Zhang et al., 2023) can be categorized as magnitude-based, one-shot pruning methods. These methods train the model using the learning objective specified in Equation (3). Upon convergence, they perform one-shot pruning based on a pruning threshold determined by the values of λ, $\sigma_{0}^{2}$, and $\sigma_{1}^{2}$. Then, the pruned model is retrained using only the loss function ℒ with the pruned parameters fixed to 0. The detailed algorithm is provided in Section S2 of the Supplement.

Their algorithms have set new standards in performance for pruning smaller models like ResNet-20, ResNet-32, and LSTMs. This highlights the effectiveness of MGP. However, translating these successes to larger, transformer-based models introduces challenges due to the many sensitive hyperparameters involved. Additionally, the retraining stage requires further tuning of hyperparameters such as learning rate and batch size, adding another layer of complexity. This is a particular concern given the computational resources required to train larger models.

Besides the aforementioned challenges, achieving a target sparsity level $v^{\left(T\right)}$ adds further complexity. In their approach, $\sigma_{1}^{2}$ and λ are held constant, while $\sigma_{0}^{2}$ is initialized to a large value, denoted as ${\left(\sigma_{0}^{\text{init}}\right)}^{2}$. This initial setting is designed to closely align the proportion of pretrained model parameters that fall below the initial pruning threshold with $v^{\left(T\right)}$. A linear scheduler then gradually reduces $\sigma_{0}^{2}$ from ${\left(\sigma_{0}^{\text{init}}\right)}^{2}$ to ${\left(\sigma_{0}^{\text{end}}\right)}^{2}$. Throughout this prior-annealing (PA) process, the MGP penalty on these parameters increases, effectively driving them toward zero, so that they can be one-shot pruned in the end. However, as explained in Section 2.2, this pruning strategy may hurt the performance of the sparsified model. We provide experimental evidence in Section 4.6 to support our arguments.

## 3 The MGPP Method

To overcome these challenges, we introduce the MGPP method, summarized in Algorithm 1. Instead of relying on annealing the MGP to shrink the parameter down to zero, which tends to fix the sparsity pattern too early in the training process, we take a different approach. We keep the MGP fixed during training and utilize the cubic sparsity scheduler to gradually prune model parameters. When a set of parameters is pruned (i.e., set to zero), they receive gradients only from the loss function in the subsequent training iteration, as the gradients from the MGP become zero. This can serve as a remedy for false selection, thereby overcoming premature pruning of critical parameters. Parameters with large gradients from the loss are more likely to escape the region with large penalties, giving them a chance to be reconsidered for pruning later. Conversely, parameters that receive smaller gradients from the loss function will likely remain within the penalized region, making them candidates for future pruning.

Our proposed algorithm introduces only three additional hyperparameters beyond the standard ones: λ, $\sigma_{0}^{2}$, and $\sigma_{1}^{2}$. A comprehensive hyperparameter-sensitivity analysis is given in Section 4.8. Briefly, we find λ to be robust and set it universally to 10^−7^. Preliminary experiments suggest that, when combined with a sparsity scheduler, it is preferable to set $\sigma_{0}^{2}$ to very small values such as 1 × 10^−10^, in contrast to previous works that often set $\sigma_{0}^{2}$ (specifically, ${\left(\sigma_{0}^{\text{end}}\right)}^{2}$) to larger values, i.e., [1 × 10^−7^, 1 × 10^−5^]. We limit $\sigma_{0}^{2}$ and $\sigma_{1}^{2}$ to the sets {1 × 10^−9^, 1 × 10^−10^} and {0.05, 0.1}, respectively. Our approach significantly reduces the computational burden associated with hyperparameter tuning, especially when it comes to training large transformer models. Despite these restrictions, our method outperforms other baselines, as demonstrated in the experimental results section (see Section 4).

We also found that gradually incorporating the MGP improves the performance of the sparsified model. This ‘prior warm-up’ can be seamlessly integrated into the warm-up phase of the sparsity scheduler, denoted by Equation 1. Let $\eta^{\left(t\right)}$ be the prior coefficient at training step t, we have

(5) v(t),η(t)={0,ttit<tiv(T)−v(T)(1−t−titf−ti)3,1ti≤t≤tfv(T),1tf<t≤T.}

In the Appendix A, we provide a loose justification for the parameter estimation consistency of the sparse transformer model with the mixture Gaussian prior, drawing upon the established theory from Liang et al. (2022) and Liang et al. (2018a). This justifies the use of the mixture Gaussian prior for sparsifying the transformer model as proposed in the paper, while the MGPP method proposed above is mainly for locating a maximum *a posteriori* (MAP) solution for the complex transformer model. Additionally, we note that the parameter estimation consistency for the sparse transformer model is subject to loss-invariant transformations. That is, the model is assumed to be unique up to loss-invariant transformations, e.g., reordering the hidden neurons of the same hidden layer or simultaneously changing the signs or scales of certain connection weights and biases. The same assumption has often been used in studying theoretical properties of deep neural network models, see e.g., Liang et al. (2018b) and Sun et al. (2022a).

**Table:** 

Algorithm 1 MGPP.
1:Input:training datasetDn,pretrained modelθ(0),number of training epochsE,mini-batchsizem,λ,σ02,σ12,ti,tf,Δt2:Initialize:t=1,T=⌈En∕m⌉,optimizer(e.g.,AdamW(Loshchilov and Hutter,2019))3:forepoch from1toEdo4:foreach mini-batchℬsampled fromDndo5:Calculate gradients of the loss through backpropagation∇θ(t−1)ℒ(ℬ,θ(t−1))6:Calculatev(t)andη(t)based on Eq.57:Calculate gradients of MGP based on Eq.4−η(t)1n∇θ(t−1)log(π(θ(t−1);λ,σ02,σ12))8:Updateθ(t−1)→θ(t)by an optimization step9:Calculate scoressS(t)=(∣θ1(t)∣,…,∣θd(t)∣)10:iftmodΔt=0ort>tfthenθj(t)={θj(t)ifSj(t)in topv(t)%0otherwise}11:endif12:Sett=t+113:endfor14:endfor

## 4 Experiments

### 4.1 Experimental Setup

The performance of the final pruned models can be influenced by various factors unrelated to the pruning methodology, including the number of training epochs, the maximum input sequence length, maximum gradient norm, and the number of beams used for evaluation in natural language generation tasks, among others. To control for these variables and ensure a fair comparison with different baselines, we follow the guidelines established in the recent works (Zhang et al., 2022; Kurtic et al., 2022; Li et al., 2023). We set all these methodology-unrelated factors to match those used in (Zhang et al., 2022; Kurtic et al., 2022; Li et al., 2023). We only tune methodology-related factors, such as λ, $\sigma_{1}^{2}$, and $\sigma_{0}^{2}$, along with standard hyperparameters like learning rate and batch size, which are also tuned in the baseline methods. Additional details are provided below and in the Section S3 of the Supplement.

We evaluate the proposed method, MGPP, across three downstream NLP tasks: natural language understanding, question answering, and natural language generation, as well as in the upstream pruning scenario. Specifically, we apply MGPP to three pretrained transformer-based language models: DeBERTaV3_base_ (180 million parameters), BERT_base_ (110 million parameters), and BART_large_ (400 million parameters).

Following the prior works (Louizos et al., 2017; Sanh et al., 2020; Zhang et al., 2022; Kurtic et al., 2022; Li et al., 2023), we prune all weight matrices, except for embeddings, LayerNorm, and the final prediction module. Our implementation is based on the publicly available Hugging Face Transformers library (Wolf et al., 2020). All performance metrics reported for MGPP are derived from the mean of five independent runs, each using a different random seed.

We compare MGPP with the following baselines:

- Gradual Magnitude Pruning (GMP) (Zhu and Gupta, 2017) is a simple yet strong magnitude-based iterative pruning baseline, widely recognized as one of the best magnitude-based pruning methods.
- Movement Pruning (MvP) (Sanh et al., 2020) is a sensitivity-based (first-order) iterative pruning method that prunes parameters based on their movement away from zero.
- Iterative pruning (ITP) (Molchanov et al., 2019) is a sensitivity-based (first-order) iterative pruning method that prunes parameters at each iteration if their importance scores fall below a hard threshold.
- PLATON (Zhang et al., 2022) is a sensitivity-based (first-order) iterative pruning method designed to capture the uncertainty of model parameters’ importance scores during the pruning process.
- oBERT (Kurtic et al., 2022) is a sensitivity-based (second-order) iterative pruning method that utilizes a diagonal block-wise approximation of the empirical Fisher matrix.
- LoSparse (Li et al., 2023) is a sensitivity-based (first-order) iterative pruning method for transformer-based language models that integrates low-rank and sparse matrices to prune weight matrices effectively.

### 4.2 Natural Language Understanding

We assess the pruning performance of MGPP on BERT_base_ (Devlin et al., 2019) and DeBERTaV3_base_ models (He et al., 2021) by conducting experiments on the General Language Understanding Evaluation (GLUE) benchmark (Wang et al., 2018), which includes a variety of tasks. Specifically, GLUE features two single-sentence classification tasks, SST-2 (Socher et al., 2013) and CoLA (Warstadt et al., 2019), as well as three tasks focused on similarity and paraphrasing: MRPC (Dolan and Brockett, 2005), STS-B (Cer et al., 2017), and QQP. Additionally, the benchmark includes four natural language inference tasks: MNLI (Williams et al., 2017), QNLI (Rajpurkar et al., 2016), RTE (Dagan et al., 2005), and WNLI (Levesque et al., 2012). In accordance with prior studies, we omit WNLI from our experiments. Additional details regarding the datasets can be found in the Section S3.1 of the Supplement.

A table containing training details, such as learning rate, batch size, the number of training epochs, $\sigma_{0}^{2}$, and $\sigma_{1}^{2}$ for each dataset, is presented in the Section S3.1 of the Supplement.

The results on the GLUE development set are summarized in Tables 1 and 2; all baseline results are directly taken from Zhang et al. (2022); Li et al. (2023). MGPP consistently achieves equal or superior performance compared to existing approaches across most datasets and sparsity levels. Notably, as the amount of training data increases, our method performs even better relative to other baselines. For instance, as shown in Table 1, at a target sparsity level of 90%, MGPP achieves 85.2/84.2% accuracy on the MNLI dataset—3.5/2.4% higher than the best-performing baseline, LoSparse. Remarkably, our results at 90% sparsity for MNLI even surpass LoSparse’s performance at 80% sparsity, demonstrating the effectiveness of our approach with more data and higher sparsity levels. Similarly, Table 2 shows that our method, while using less memory, achieves better or comparable results to PLATON.

### 4.3 Question Answering

We assess the performance of MGPP on the DeBERTaV3_base_ model (He et al., 2021) by conducting experiments on a standard question answering dataset SQuADv1.1 (Rajpurkar et al., 2016). SQuADv1.1 is a reading comprehension benchmark consisting of questions derived from Wikipedia articles, with 88k training samples and 10k validation samples.

For all sparsity levels, the number of training epochs and batch sizes is set to 10 and 16, respectively. We set the learning rate to 5 × 10^−5^, and for the MGP, we specify $\sigma_{0}^{2}=1\times 10^{-10}$ and $\sigma_{1}^{2}=0.05$. More details are given in the Section S3.2 of the Supplement.

The results on the SQuADv1.1 validation set are summarized in Table 3 using two performance metrics: exact match (EM) and F1. All baseline results are taken directly from (Li et al., 2023). MGPP demonstrates performance that is either superior to or on par with existing methods across all sparsity levels.

Consistent with our findings on the GLUE benchmark, our method is especially effective in high sparsity regimes. For example, at the 90% sparsity level, MGPP outperforms LoSparse (the best-performing baseline) by 5.1% in terms of EM.

### 4.4 Natural Language Generation

We assess the pruning performance of MGPP on the BART_large_ model (Lewis et al., 2019) by conducting experiments on two natural language generation datasets: XSum (Narayan et al., 2018) and CNN/DailyMail (Hermann et al., 2015). The objective is to generate either a concise summary or a highlight that captures the main point of a document. Refer to the Section S3.3 of the Supplement for more detailed information about the datasets.

For all sparsity levels and both datasets, we set the number of training epochs to 12 and the batch size to 32. The beam search length is fixed at 8, and the learning rate is set to 2 × 10^−5^. For the MGP, we set $\sigma_{0}^{2}=1\times 10^{-10}$ and $\sigma_{1}^{2}=0.1$. Additional details can be founded in the Section S3.3 of the Supplement.

The results on the test sets of both datasets are summarized in Table 4, using three performance metrics: ROUGE 1/2/Lsum scores (Lin, 2004). All baseline results are directly adopted from (Li et al., 2023). The comparison indicates that MGPP outperforms existing approaches across all sparsity levels on both datasets. Notably, the larger the performance gap between the fully fine-tuned dense model and its sparsified counterpart, the greater the extent to which MGPP outperforms the baselines. This trend is especially pronounced for the XSum dataset, where the higher task complexity leads to a more significant gap.

### 4.5 Upstream Pruning

Upstream pruning (Zafrir et al., 2021) provides an alternative to the conventional downstream pruning approach. In upstream pruning, the model is pruned during the semi-supervised pre-training phase and then fine-tuned sparsely on specific downstream tasks. Models pruned in this manner generally exhibit improved generalization capabilities (Chen et al., 2020; Zafrir et al., 2021) and require significantly fewer computational resources for fine-tuning, as only the remaining parameters need to be adjusted. However, upstream pruning typically demands a considerably larger dataset compared to downstream pruning. Currently, oBERT (Kurtic et al., 2022) stands as the state-of-the-art method for upstream pruning on BERT-like models and serves as the primary baseline for comparison in this section.

Following the guidelines established by oBERT, we use the BERT_base_ model fine-tuned on two upstream datasets: BookCorpus and English Wikipedia. We then apply MGPP to prune the model on the same datasets for 3 epochs. Finally, we sparse-fine-tune the pruned model on the GLUE benchmark for 8 epochs. Detailed hyperparameters are provided in Section S3.4 of the Supplement.

The results are presented in Table 5. We adopt all baseline results directly from Kurtic et al. (2022). Notably, MGPP outperforms oBERT, despite the latter’s additional memory requirement of O(50d) and its greater computational complexity of O(mBd).

### 4.6 Ablation Study

To justify the contributions of various components and design choices in our method, we conduct an ablation study in this section. We compare our approach to Prior-Annealing (PA) (Sun et al., 2021; Zhang et al., 2023), which provides an effective implementation for sparsifying deep neural network models with the MGP prior in the one-shot pruning style.

Additionally, we replace the MGP in our method with an $L_{2}$ penalty (denoted as $L_{2}$) to confirm the significance of this particular prior. As we have previously discussed, the MGP imposes penalties on parameters in a way that is similar to $L_{2}$ regularization on a larger scale of the parameter space.

The ablation study is carried out on the DeBERTaV3_base_ model on three datasets from the GLUE benchmark: MNLI, MRPC, and SST-2. These datasets represent diverse task categories, including single-sentence classification, similarity and paraphrasing, and natural language inference. They also vary in training set size, ranked from large to small: MNLI, SST-2, MRPC.

The results are summarized in Table 6. Notably, we performed extensive hyperparameter search for PA to ensure a fair comparison (details are given in Section 3.5 of the Supplement). Despite this effort, MGPP consistently outperforms PA on all three datasets and across all sparsity levels. When compared to $L_{2}$, the advantage of MGPP becomes increasingly pronounced as sparsity increases. For example, on the MNLI dataset, at 80% sparsity, MGPP surpasses $L_{2}$ by 1.2/1.3%. At 90% sparsity, this margin grows significantly, with MGPP outperforming $L_{2}$ by 3.6/3.0%.

### 4.7 Algorithm Analysis

**Figure 2:**
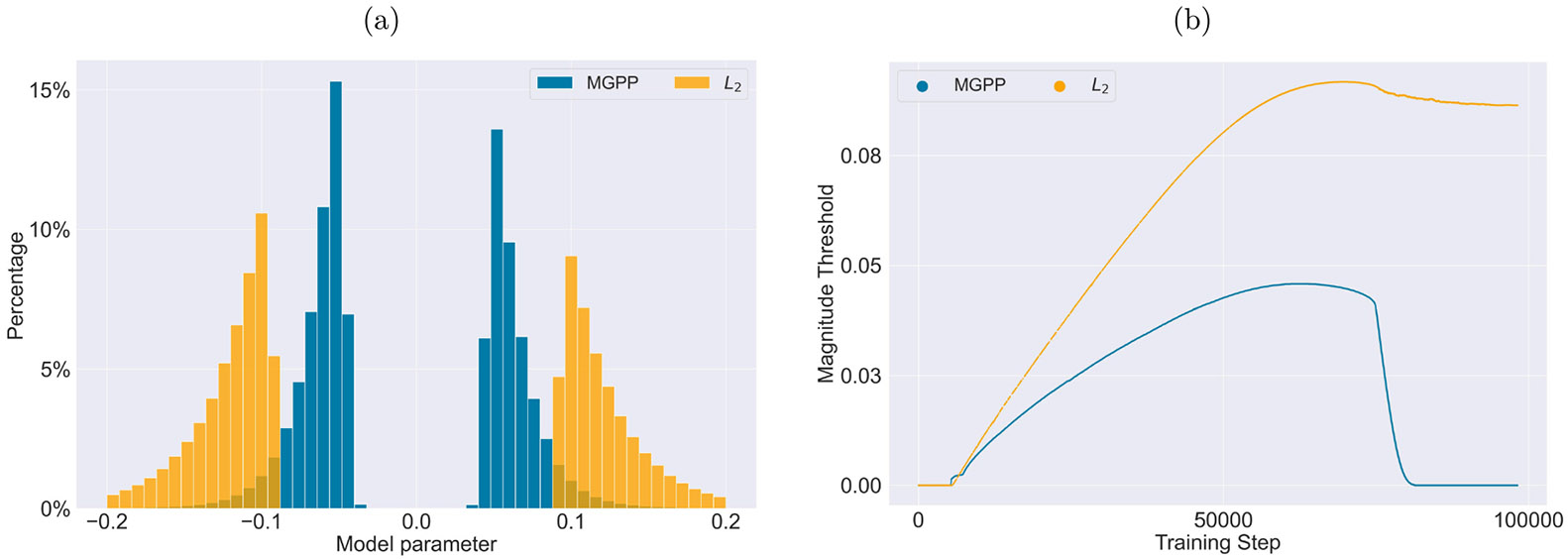
A comparative analysis of MGPP and $L_{2}$: (a) distributions of remaining nonzero parameters, (b) magnitude pruning thresholds during training.

To better illustrate the impact of MGP, Figure 2 depicts the distribution of remaining nonzero parameters and the evolution of pruning thresholds during training for a 90% sparsified DeBERTaV3_base_ model on the MNLI dataset, comparing our method against the $L_{2}$ variant. We observe that both MGPP and $L_{2}$ tend to prune parameters that are close to zero. However, as shown in Figure 2(b), the spike component in the MGP more effectively drives parameters toward zero, resulting in a lower pruning threshold. In contrast, $L_{2}$ fails to similarly reduce the pruning threshold, leading to a performance gap in generalization.

### 4.8 Hyperparameter Sensitivity Analysis

The proposed method, MGPP, introduces six hyperparameters: three from the Mixture Gaussian Prior (MGP) and three from the cubic sparsity scheduler. It is important to note that the cubic sparsity scheduler is also employed by the baselines considered in this work, so no additional hyperparameters are introduced when comparing to these baselines.

In this section, we focus on the sensitivity of the three MGP-specific hyperparameters:

- λ: MGPP is robust to this hyperparameter, which we fix at 1 × 10^−7^ in all experiments. Changing λ only slightly adjusts the width of the spike component (see Section S1 of the Supplement). Preliminary experiments show that its impact on performance is negligible, and a value below 0.1 is generally sufficient.
- $\sigma_{0}^{2}$: A general guideline is to use a smaller value when more training samples are available. We limited our selection to the set {1 × 10^−9^, 1 × 10^−10^}. Values in the range $\sigma_{0}^{2}\leq 1\times 10^{-8}$ do not significantly affect performance.
- $\sigma_{1}^{2}$: Similar to $\sigma_{0}^{2}$, smaller values are recommended for larger datasets. We restricted our choices to the set {0.1, 0.05}. Although this hyperparameter has more impact on performance, the suggested values work well across all experiments.

For more detailed discussion on how these hyperparameters shape the prior landscape, please refer to Section S1 of the Supplement.

## 5 Conclusion

In this paper, we have developed MGPP, a novel magnitude-based iterative pruning method designed to sparsify large-scale transformer models. Extensive experimental results on various natural language processing tasks and two transformer-based language models demonstrate the effectiveness and efficiency of MGPP, particularly in settings with abundant training data or high sparsity. Additionally, we provided a theoretical justification for the consistency of MGPP, offering insights into its strong performance.

Transformer model pruning is an ongoing research area. Besides the pruning scores discussed in Section 2.1, more complex pruning scores have also been proposed in the literature. For instance, the Platon method (Zhang et al., 2022) prunes the model based on the upper confidence bound of the weight importance, while the WoodFisher (Singh and Alistarh, 2020), M-FAC (Frantar et al., 2021), and oBERT (Kurtic et al., 2022) methods utilize Hessian-based information for pruning. These pruning scores can also be computed with the mixture Gaussian prior, leading to new variants of the proposed method. Notably, the consistency property of the MGPP method can be extended to these new variants, providing a theoretical guarantee for their validity. In contrast, existing methods often lack such theoretical support for their performance. Additionally, we note that the calculation of complex pruning scores often requires higher GPU memory than that needed for magnitude-based pruning scores.

While model compression often involves other strategies like knowledge distillation and quantization, these are not mutually exclusive with pruning. For instance, one could enhance the performance of a pruned model through knowledge distillation and further reduce storage requirements by quantizing the remaining parameters. We leave such extensions for future work.

## Supplementary Material

Supplementary Material

The supplementary material includes (i) a brief description for the prior annealing algorithm, (ii) detailed experimental settings, and (iii) a folder (code) which contains all the code for the proposed algorithm MGPP as well as the code to reproduce the experiments.

## Figures and Tables

**Table 1:** Comparison of different pruning methods for the DeBERTaV3_base_ model on the GLUE development sets, where “N.A.” indicates non-convergence of the model, “m/mm” denotes the accuracy for the matched and mismatched development sets of the MNLI task, and other metrics (i.e., Acc, F1, Mcc, P/S Corr) are defined in Table S1 (in the Supplement). The highest-performing results for each dataset are highlighted in bold.

Sparsity	Method	MNLI m/mm	RTE Acc	QNLI Acc	MRPC Acc/F1	QQP Acc/F1	SST-2 Acc	CoLA Mcc	STS-B P/S Corr
**0%**	DeBERTaV3_base_	90.5/90.6	82.0	94.0	89.5/93.3	92.4/89.8	95.3	69.2	91.6/91.1
**80%**	MvP	N.A.	61.2	86.0	79.2/85.0	N.A.	89.4	N.A.	84.3/84.3
	ITP	82.8/82.5	N.A.	87.8	82.0/87.0	90.0/86.4	90.8	49.0	87.4/87.0
	LoSparse	84.5/83.8	68.0	88.6	85.0/89.4	90.6/87.2	91.7	50.0	88.8/88.5
	MGPP	**87.2/86.9**	**70.0**	**91.7**	**85.5**/89.4	**91.3/88.3**	**93.2**	**56.3**	**88.9/88.5**
**85%**	MvP	N.A.	59.0	N.A.	78.5/84.3	N.A.	89.0	N.A.	83.9/83.9
	ITP	81.7/81.3	N.A.	85.4	80.5/86.3	89.1/85.2	89.3	45.8	86.8/86.3
	LoSparse	83.3/82.9	66.9	87.6	83.6/88.0	90.3/87.0	90.4	46.8	87.7/87.3
	MGPP	**86.0/85.9**	**68.3**	**90.9**	**84.3/88.7**	**91.1/87.9**	**92.3**	**50.9**	**88.0/87.5**
**90%**	MvP	N.A.	N.A.	N.A.	77.0/83.4	N.A.	88.0	N.A.	N.A.
	ITP	79.7/79.6	N.A.	82.3	78.5/84.3	88.3/84.4	88.3	38.0	86.3/86.0
	LoSparse	81.7/81.8	66.0	86.1	82.3/**87.4**	89.5/86.0	89.2	40.0	87.2/**87.0**
	MGPP	**85.2/84.2**	**66.2**	**88.8**	**82.6**/87.1	**91.1/88.0**	**90.2**	**48.0**	87.2/86.7

**Table 2:** Comparison of different methods for the BERT_base_ model on the GLUE development sets in downstream tasks, where “m/mm” denotes the accuracy for the matched and mismatched development sets of the MNLI task. Refer to Table S1 (in the Supplement) for the other metrics used in the table.

Sparsity	Method	MNLI m/mm	QQP Acc/F1	QNLI Acc	SST-2 Acc
**0%**	BERT_base_	84.6 / 83.4	91.5 / 88.5	91.3	92.7
**80%**	GMP	81.5 / 82.9	86.0 / 83.8	89.2	84.3
	MvP	81.6 / 82.1	90.6 / 87.5	88.3	89.0
	PLATON	83.1 / 83.4	90.7 / 87.5	90.1	91.5
	MGPP	83.1 / 83.4	**90.8** / **87.6**	**90.2**	**91.9**
**90%**	GMP	78.8 / 79.0	78.8 / 77.0	86.6	80.7
	MvP	80.7 / 81.1	90.2 / 86.7	86.6	87.4
	PLATON	82.0 / 82.2	90.2 / 86.8	88.9	90.5
	MGPP	**82.1** / 82.2	**90.4** / **87.1**	**89.2**	**90.8**

**Table 3:** Comparison of MGPP, ITP, and LoSparse for the DeBERTaV3_base_ model on the SQuADv1.1 validation set, where the best results for each dataset are highlighted in bold.

Dataset	SQuADv1.1 EM/F1					
Sparsity	95%	90%	80%	70%	60%	50%
DeBERTaV3_base_	87.7/93.5					
- ITP	65.2/76.1	70.9/80.3	75.0/83.9	78.2/86.2	78.2/86.2	81.5/89.6
- LoSparse	69.3/79.1	72.9/82.8	76.8/85.8	80.2/88.0	82.1/89.4	82.3/90.3
- MGPP	**73.7/82.9**	**78.0/86.2**	**80.2/88.6**	**81.1/89.5**	82.1/**90.1**	**82.5**/90.3

**Table 4:** Comparison of MGPP, ITP, and LoSparse for the BART_large_ model on the datasets: XSum and CNN/DailyMail, where “Lead-3” represents choosing the first 3 sentences as the summary, and the best results for each dataset are highlighted in bold.

Sparsity	Method	XSum	CNN/DailyMail
**0%**	Lead-3	16.30/1.60/11.95	40.42/17.62/36.67
	BART_large_	45.14/22.27/37.25	44.16/21.28/40.90
**50%**	ITP	38.42/16.32/31.43	40.76/18.30/37.65
	LoSparse	39.18/16.91/31.62	41.54/19.04/38.58
	MGPP	**42.92/19.70/34.80**	**42.59/19.90/39.57**
**60%**	ITP	36.71/14.96/29.86	40.52/18.10/37.31
	LoSparse	38.30/16.02/30.72	41.42/19.00/38.47
	MGPP	**41.69/18.75/33.69**	**42.27/19.63/39.26**
**70%**	ITP	34.42/13.15/27.99	40.35/17.98/37.15
	LoSparse	37.41/15.42/30.02	41.21/18.84/38.21
	MGPP	**40.20/17.33/32.34**	**41.93/19.21/38.88**

**Table 5:** Comparison of MGPP and oBERT on development sets for the upstream-pruned model BERT_BASE_ at the 90% sparsity level, where “m/mm” indicates the accuracy for the matched and mismatched development sets of the MNLI task.

Sparsity	Method	MNLI m/mm	QNLI Acc	QQP Acc/F1	SST-2 Acc
**0%**	BERT_BASE_	84.6 / 83.4	91.3	91.5 / 88.5	92.7
**90%**	oBERT	82.2 / 82.5	89.3	90.4 / 87.1	92.0
	MGPP	**82.4 / 82.6**	**89.8**	**90.5 / 87.3**	**92.3**

**Table 6:** Comparison of MGPP with two ablation variants, PA and $L_{2}$, on the MNLI, MRPC, and SST-2 datasets, where the results of MGPP are taken from Table 1.

Sparsity	Method	MNLI m/mm	MRPC Acc/F1	SST-2 Acc
**80%**	PA	83.8/82.9	83.1/88.3	90.1
	$L_{2}$	86.0/85.6	82.4/87.3	91.5
	MGPP	**87.2/86.9**	**85.5/89.4**	**93.2**
**85%**	PA	81.6/81.4	78.4/85.7	88.5
	$L_{2}$	83.8/84.6	76.5/82.0	90.5
	MGPP	**86.0/85.9**	**84.3/88.7**	**92.3**
**90%**	PA	79.5/78.9	77.6/83.8	87.2
	$L_{2}$	81.6/81.2	71.8/82.1	87.1
	MGPP	**85.2/84.2**	**82.6/87.1**	**90.2**

## Appendix

### A Consistency of Sparse Transformer with the MGP Penalty

The appendix is organized as follows. Section A provides a loose justification for the consistency of the proposed MGPP method. In Section A.1, we introduce an auxiliary stochastic transformer model; and in Section A.2, we provide a constructive proof for the consistency of the sparse stochastic transformer estimator under the theoretical framework of the imputation regularized-optimization (IRO) algorithm (Liang et al., 2018a). With this preparation, we then establish the consistency of the sparse transformer model with a mixture Gaussian prior/penalty.

#### A.1 Asymptotic Equivalence Between the Transformer and Stochastic Transformer Models

The asymptotic equivalence between the transformer and stochastic transformer has been studied in Kim et al. (2024). To make the paper self-contained, we provided a brief review as follows.

##### Transformer Model

Following Thickstun (2020), we define a transformer block as follows.

Let $x={\left(x_{1},x_{2},\ldots ,x_{n}\right)}^{T}\in R^{n\times d}$ denote an input matrix to a transformer block, which transforms x to $z\in R^{n\times d}$ with the detail specified as follows:

(6)
$$Q^{\left(h\right)}(x_{i})=W_{h,q}^{T}x_{i},\;K^{\left(h\right)}(x_{i})=W_{h,k}^{T}x_{i},\;V^{\left(h\right)}(x_{i})=W_{h,v}^{T}x_{i},\;W_{h,q},W_{h,k},W_{h,v}\in R^{d\times k},\;\alpha_{i,j}^{\left(h\right)}={\text{softmax}}_{j}\left(\frac{\langle Q^{\left(h\right)}(x_{i}),K^{\left(h\right)}(x_{j})\rangle }{\sqrt{k}}\right),\;i,j=1,2,\ldots ,n,\;u_{i}=\sum_{h=1}^{H}W_{c,h}^{T}\sum_{j=1}^{n}\alpha_{i,j}^{\left(h\right)}V^{\left(h\right)}(x_{j}),\;W_{c,h}\in R^{k\times d},\;{\tilde{u}}_{i}=\text{LayerNorm}(x_{i}+u_{i};\gamma_{1},\beta_{1}),\;\gamma_{1},\beta_{1}\in R^{d},\;{\tilde{z}}_{i}=W_{2}^{T}\text{ReLU}(W_{1}^{T}{\tilde{u}}_{i}),\;W_{1}\in R^{d\times m},\;W_{2}\in R^{m\times d},\;z_{i}=\text{LayerNorm}({\tilde{u}}_{i}+{\tilde{z}}_{i};\gamma_{2},\beta_{2}),\;\gamma_{2},\beta_{2}\in R^{d},$$

where H denotes the number of attention heads, 〈⋅,⋅〉 denotes inner product, and the layerNorm is given by

$$\text{LayerNorm}(\alpha ;\gamma ,\beta )=\gamma ⊙\frac{\left(a-\bar{a}1_{d}\right)}{s_{a}}+\beta ,$$

where ⊙ is the element-wise multiplication operator, $a={\left(a_{1},a_{2},\ldots ,a_{d}\right)}^{T}\in R^{d}$, $1_{d}$ is an d-vector of ones, $\bar{a}=\frac{1}{d}\sum_{i=1}^{d}a_{i}$, and $s_{a}=\sqrt{\frac{1}{d}\sum_{i=1}^{d}{\left(a_{i}-\bar{a}\right)}^{2}}$. For convenience, we denote the transformer block by the function $f_{\theta }:R^{n\times d}\to R^{n\times d}$, where the parameters θ consist of {$W_{h,q}$, $W_{h,k}$, $W_{h,v}$, $W_{c,h}:h=1,2,\ldots ,H$} and {$\gamma_{1}$, $\beta_{1}$, $\gamma_{2}$, $\beta_{2}$, $W_{1}$, $W_{2}$}. A transformer is a composition of L transformer blocks: $f_{\theta_{L}}\circ \cdots \circ f_{\theta_{1}}(x)\in R^{n\times d}$, each block has its own parameters. The common settings of the hyperparameters are d=512, k=64, m=2048, and H=8.

##### Stochastic Transformer Model

The stochastic transformer model is defined as follows:

(7)
$$Q^{\left(h\right)}(x_{i})=W_{h,q}^{T}x_{i}+\epsilon^{h,q},\;K^{\left(h\right)}(x_{i})=W_{h,k}^{T}x_{i}+\epsilon^{h,k}\;V^{\left(h\right)}(x_{i})=W_{h,v}^{T}x_{i}+\epsilon^{h,v}\;W_{h,q},W_{h,k},W_{h,v}\in R^{d\times k},\;\alpha_{i,j}^{\left(h\right)}={\text{softmax}}_{j}\left(\frac{\langle Q^{\left(h\right)}(x_{i}),K^{\left(h\right)}(x_{j})\rangle }{\sqrt{k}}\right),\;i,j=1,2,\ldots ,n,\;u_{i}=\sum_{h=1}^{H}W_{c,h}^{T}\sum_{j=1}^{n}\alpha_{i,j}^{\left(h\right)}V^{\left(h\right)}(x_{j})+\epsilon^{i,u},\;W_{c,h}\in R^{k\times d},\;{\tilde{u}}_{i}=\text{LayerNorm}(x_{i}+u_{i};\gamma_{1},\beta_{1}),\;\gamma_{1},\beta_{1}\in R^{d},\;{\tilde{z}}_{i}=W_{2}^{T}\text{ReLU}(W_{1}^{T}{\tilde{u}}_{i})+\epsilon^{i,\tilde{z}},\;W_{1}\in R^{d\times m},\;W_{2}\in R^{m\times d},\;z_{i}=\text{LayerNorm}({\tilde{u}}_{i}+{\tilde{z}}_{i};\gamma_{2},\beta_{2}),\;\gamma_{2},\beta_{2}\in R^{d},$$

where the noise variables $\epsilon^{h,q}∼N(0,\sigma_{q}^{2}I_{k})$, $\epsilon^{h,k}∼N(0,\sigma_{k}^{2}I_{k})$, $\epsilon^{h,v}∼N(0,\sigma_{v}^{2}I_{k})$, $\epsilon^{i,u}∼N(0,\sigma_{u}^{2}I_{d})$, and $\epsilon^{i,\tilde{z}}∼N(0,\sigma_{\tilde{z}}^{2}I_{d})$ are mutually independent. Note that $\sigma_{q}^{2}$, $\sigma_{k}^{2}$, $\sigma_{v}^{2}$, $\sigma_{u}^{2}$, and $\sigma_{\tilde{z}}^{2}$ are all known, pre-specified by user. As a consequence of introducing the noise variables, we can treat $Q^{\left(h\right)}’s$, $K^{\left(h\right)}’s$, $V^{\left(h\right)}’s$, $u_{i}’s$, and ${\tilde{z}}_{i}’s$ as latent variables, and decompose the model as

(8)
$$\pi_{\theta }(z,Q,K,V,U,\tilde{Z}|x)=\prod_{h=1}^{H}\pi (Q^{\left(h\right)}|x,\theta^{\left(1\right)})\prod_{h=1}^{H}\pi (K^{\left(h\right)}|x,\theta^{\left(2\right)})\prod_{h=1}^{H}\pi (V^{\left(h\right)}|x,\theta^{\left(3\right)})\;\;\times \pi (U|x,\theta^{\left(4\right)},Q,K,V)\pi (\tilde{Z}|x,\theta^{\left(5\right)},Q,K,V,U)\pi (z|Q,K,V,U,\tilde{Z},x,\theta^{\left(6\right)}),$$

where $Q=\{Q^{\left(1\right)},Q^{\left(2\right)},\ldots ,Q^{\left(H\right)}\}$, $K=\{K^{\left(1\right)},K^{\left(2\right)},\ldots ,K^{\left(H\right)}\}$, $V=\{V^{\left(1\right)},V^{\left(2\right)},\ldots ,V^{\left(H\right)}\}$, $U=\{u_{1},u_{2},\ldots ,u_{n}\}$, $\tilde{Z}=\{{\tilde{z}}_{1},{\tilde{z}}_{2},\ldots ,{\tilde{z}}_{n}\}$, $\theta^{\left(1\right)}=\{W_{h,q}:h=1,2,\ldots ,H\}$, $\theta^{\left(2\right)}=\{W_{h,k}:h=1,2,\ldots ,H\}$, $\theta^{\left(3\right)}=\{W_{h,v}:h=1,2,\ldots ,H\}$, $\theta^{\left(4\right)}=\{W_{c,h}:h=1,2,\ldots ,H\}$, $\theta^{\left(5\right)}=\{W_{1},W_{2},\gamma_{1},\beta_{1}\}$, and $\theta^{\left(6\right)}=\{\gamma_{2},\beta_{2}\}$.

The asymptotic equivalence between the transformer and stochastic transformer models have been established in Kim et al. (2024), where it was shown that the two models have asymptotically the same loss function under appropriate conditions.

More precisely, they showed that there exists a small value τ(d,k,m,H), as a function of d, k, m and H, such that

(9)
$$\underset{\theta \in \Theta }{\text{sup}}\frac{1}{n}|\;\log\;\pi_{\theta }(z,Q,K,V,U,\tilde{Z}|x)-\log\;{\tilde{\pi }}_{\theta }(z|x)\;|\overset{\;p}{\to }0,$$

as n→∞ and $\text{max}\{\sigma_{q},\sigma_{k},\sigma_{v},\sigma_{u},\sigma_{\tilde{z}}\}≺\tau (d,k,m,H)$, where $\pi_{\theta }(z,Q,K,V,U,\tilde{Z}|x)$ represents the pseudo-complete data likelihood function of the stochastic transformer by treating Q, K, V, U, and $\tilde{Z}$ as latent variables, ${\tilde{\pi }}_{\theta }(z|x)$ represents the likelihood function of the transformer, and $\overset{\;p}{\to }$ denotes convergence in probability. We note that similar techniques have been used in Liang et al. (2022) and Sun and Liang (2022) in establishing the asymptotic equivalence between the deep neural network and stochastic neural network (StoNet) models.

#### A.2 Consistency of Sparse Transformer

By treating {Q, K, V, U, $\tilde{Z}$} as latent variables, the parameters θ of the stochastic transformer model can be estimated using a regularization approach as follows:

(10)
$${\hat{\theta }}_{n}=\text{arg}\max_{\theta }\left\{\;\log\;\pi_{\theta }(z,Q,K,V,U,\tilde{Z}|x)+\log\;P_{\lambda }(\theta )\;\right\},$$

where $P_{\lambda }(\theta )$ denotes the sparsity penalty imposed on θ, and λ is the tuning parameter. With an appropriate choice of $P_{\lambda }(\theta )$, we can provide a constructive proof for the consistency of ${\hat{\theta }}_{n}$ based on the IRO algorithm (Liang et al., 2018a).

The IRO algorithm starts with an initial weight setting ${\hat{\theta }}_{n}^{\left(0\right)}$ and then iterates between the imputation and regularized optimization steps:

- **Imputation:** For each block, conditioned on the current parameter estimate $\theta_{t-1}$, simulate the latent variables (Q, K, V, U, $\tilde{Z}$) from the predictive distribution
  (11)
  $$\pi_{\theta_{t-1}}(Q_{t},K_{t},V_{t},U_{t},{\tilde{Z}}_{t}|x,z)\propto \prod_{h=1}^{H}\pi (Q_{t}^{\left(h\right)}|x,\theta_{t-1}^{\left(1\right)})\prod_{h=1}^{H}\pi (K_{t}^{\left(h\right)}|x,\theta_{t}^{\left(1\right)})\prod_{h=1}^{H}\pi (V_{t}^{\left(h\right)}|x,\theta_{t-1}^{\left(2\right)})\;\;\times \pi (U_{t}|x,\theta_{t-1}^{\left(4\right)},Q_{t},K_{t},V_{t})\pi ({\tilde{Z}}_{t}|x,\theta_{t-1}^{\left(5\right)},Q_{t},K_{t},V_{t},U_{t})\pi (z|Q_{t},K_{t},V_{t},U_{t},{\tilde{Z}}_{t},x,\theta_{t-1}^{\left(6\right)}),$$
  where t indexes iterations, $Q_{t}=\{Q_{t}^{\left(h\right)}:h=1,2,\ldots ,H\}$, $K_{t}=\{K_{t}^{\left(h\right)}:h=1,2,\ldots ,H\}$, $V_{t}=\{V_{t}^{\left(h\right)}:h=1,2,\ldots ,H\}$, $U_{t}=\{u_{1,t},u_{2,t},\ldots ,u_{n,t}\}$, ${\tilde{Z}}_{t}=\{{\tilde{z}}_{1,t},{\tilde{z}}_{2,t},\ldots ,{\tilde{z}}_{n,t}\}$. Here, $u_{i,t}$ and ${\tilde{z}}_{i,t}$ denote, respectively, the imputed values for $u_{i}$ and $z_{i}$ at iteration t.
- **Regularized optimization:** Given the pseudo-complete data {$Q_{t}$, $K_{t}$, $V_{t}$, $U_{t}$, ${\tilde{Z}}_{t}$, z, x}, update ${\hat{\theta }}_{n}^{\left(t-1\right)}$ by maximizing the penalized log-likelihood function as follows:
  (12)
  $${\hat{\theta }}_{n}^{\left(t\right)}=\text{arg}\max_{\theta }\left\{\;\log\;\pi_{\theta }(z,Q_{t},K_{t},V_{t},U_{t},{\tilde{Z}}_{t}|x)+\log\;P_{\lambda }(\theta )\;\right\},$$
  which, by the decomposition (8), can be reduced to solving for $\theta^{\left(1\right)},\ldots ,\theta^{\left(6\right)}$, separately. The penalty function $P_{\lambda }(\theta )$ should be chosen such that ${\hat{\theta }}_{n}^{\left(t\right)}$ forms a consistent estimator for the working parameter
  $$\theta_{*}^{\left(t\right)}=\text{arg}\max_{\theta }E_{{\hat{\theta }}^{\left(t-1\right)}}\;\log\;\pi (Z,Q_{t},K_{t},V_{t},U_{t},{\tilde{Z}}_{t}|\theta ,x)\;\;=\text{arg}\max_{\theta }\int \log\;\pi (z,Q_{t},K_{t},V_{t},U_{t},{\tilde{Z}}_{t}|\theta ,x)\;\;\times \pi (Q_{t},K_{t},V_{t},U_{t},{\tilde{Z}}_{t}|z,x,\theta_{n}^{\left(t-1\right)})\pi (z|x,\theta^{*})dQ_{t}dK_{t}dV_{t}dz,$$
  where $\theta^{*}$ is defined by
  (13)
  $$\theta^{*}=\text{arg}\max_{\theta }E\;\log\;\pi (z|\theta ,x),$$
  and it corresponds to the true parameters of the underlying sparse transformer model (6).

For the sake of theoretical simplicity, we can assume that the hyperparameters of the transformer, namely, d, k, m and H, can increase with n but at a low order. By standard statistical estimation theory, see e.g., Portnoy (1988), we can achieve consistency of ${\hat{\theta }}_{n}^{\left(t\right)}$ with the mixture Gaussian prior/penalty at each iteration of the IRO algorithm.

The above assumption can be much relaxed. For example, we may assume that d, k, m and H increase with n exponentially. Under this extended assumption, we can still achieve consistency of ${\hat{\theta }}_{n}^{\left(t\right)}$ with the mixture Gaussian prior/penalty at each iteration of the IRO algorithm. This is possible by leveraging the theories presented in Song and Liang (2022), Sun et al. (2022a), and Sun et al. (2021). To elaborate, the estimation of $\theta^{\left(1\right)},\ldots ,\theta^{\left(4\right)}$ is reduced to solving a series of high-dimensional linear regressions, for which consistency with the mixture Gaussian prior can be maintained, as per the theory from Song and Liang (2022). The estimation of $\theta^{\left(5\right)}$ is reduced to solving a sparse deep neural network model with the ReLU and linear activation functions, ensuring consistency with the mixture Gaussian prior based on the theories outlined in Sun et al. (2022a) and Sun et al. (2021). The case of $\theta^{\left(6\right)}$ is similar to $\theta^{\left(5\right)}$, it is reduced to solving a sparse deep neural network model when a fully connected neural network is added to connect z and y. Otherwise, the parameters {$\gamma_{2}$, $\beta_{2}$} can be uniquely determined. Note that, as mentioned in the main text, the parameters in the LayerNorm transformation are not sparsified.

Furthermore, according to Theorem 4 of Liang et al. (2018a), the estimator ${\hat{\theta }}_{n}^{\left(t\right)}$ is consistent when both n and t are sufficiently large. In summary, under mild regularity conditions and the mixture Gaussian prior, we can establish that

(14)
$$\|{\hat{\theta }}_{n}^{\left(t\right)}-\theta^{*}\|\overset{\;p}{\to }0,$$

for sufficiently large n and sufficiently large t and almost every dataset {x, y} for the stochastic transformer model.

Finally, based on (9) and under certain regularity conditions as given in Liang et al. (2022), we also have

(15)
$$\|{\tilde{\theta }}_{n}^{\left(t\right)}-\theta^{*}\|\overset{\;p}{\to }0,$$

where ${\tilde{\theta }}_{n}^{\left(t\right)}$ is a sparse transformer estimator give by

(16)
$${\tilde{\theta }}_{n}^{\left(t\right)}=\text{arg}\max_{\theta }\left\{\;\log\;f_{\theta }(z|x)+\log\;P_{\lambda }(\theta )\;\right\}.$$

In summary, through the introduction of an auxiliary stochastic transformer model and the utilization of the IRO convergence theory, we have justified the consistency of the sparse transformer model under mild regularity conditions similar to those given in Liang et al. (2022) and Liang et al. (2018a).

Finally, we note that the above justification for the consistency of the sparse transformer model is based on the assumption that $x\in R^{n\times d}$ consists of n i.i.d observations. In practice, the observations might exhibit correlations. Nevertheless, this should not significantly impact the validity of our results, as long as x contains a sufficiently large number of independent samples.
