# Supplementary material for: Magnitude Pruning of Large Pretrained Transformer Models with a Mixture Gaussian Prior
Source: J Data Sci. Author manuscript; Available in PMC 2025 Nov 20. (PMC12629628; doi:10.6339/24-jds1156)
Supplement: Supplementary Material [file NIHMS2095843-supplement-Supplementary_Material.pdf]

Journal of Data Science 0 (0), 1–7
July 2024
DOI: 0000
Research Article

Supplement for "Magnitude Pruning of Large Pretrained Transformer Models with a Mixture Gaussian Prior"

Mingxuan Zhang[1], Yan Sun[2], and Faming Liang[1,*]

[1] Department of Statistics, Purdue University, West Lafayette, IN 47907, USA
[2] Department of Biostatistics, Epidemiology, and Informatics, University of Pennsylvania, Pennsylvania, PA 19104, USA

The supplementary material is organized as follows. Section S1 provides visualization of how λ, σ_0^2, and σ_1^2 affect the landscape of the MGP. Section S2 gives a brief description of the prior annealing algorithm. Section S3 provides settings for the experiments.

S1 Mixture Gaussian Priors

In this section, we illustrate and visualize how changes in λ, σ_0^2, and σ_1^2 influence the landscape of the MGP. As shown in Figure S1 (a), the effect of λ primarily impacts the spike component of the MGP. The larger the value of λ, the wider the spike component becomes. Based on Figure S1 (b), the influence of σ_0^2 is most noticeable on the parameter space near zero. A smaller value of σ_0^2 results in a greater penalty applied to parameters within the spike component while making it smaller. Conversely, as depicted in Figure S1 (c), the impact of σ_1^2 is mainly observed in larger-scale areas. A smaller value of σ_1^2 imposes a higher penalty on parameters at a larger scale.

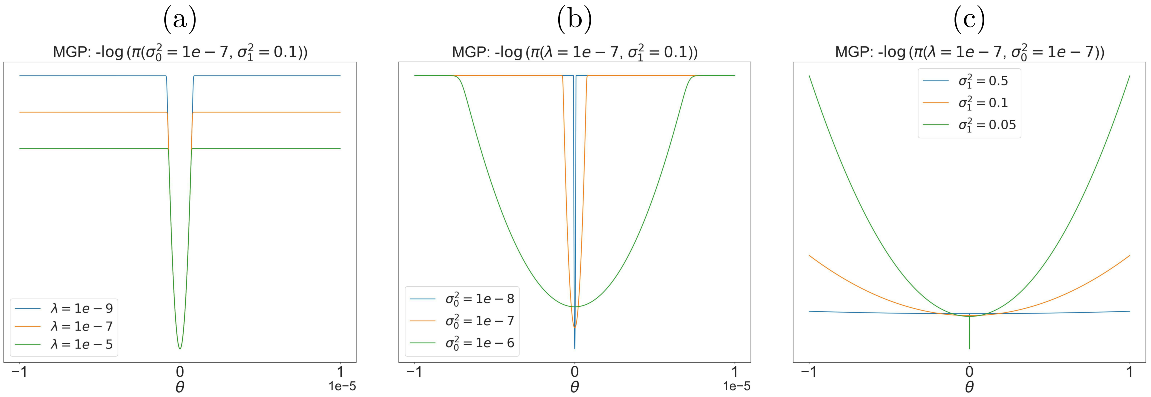

Figure S1: How λ, σ_0^2, and σ_1^2 change the landscape of the MGP.

S2 Prior Annealing

In this section, we provide a brief overview of how the Prior-Annealing (PA) algorithm (Sun et al., 2021; Zhang et al., 2023) is employed for model pruning. The detailed steps are outlined in Algorithm S1.

*Corresponding author. Email: fmliang@purdue.edu.

Algorithm S1 Prior-Annealing (PA)

1: **Input:** training dataset D_n, pretrained model $\boldsymbol{\theta}^{(0)}$, number of training epochs E, mini-batch size m, λ, σ_1^2, $(\sigma_0^{\text{init}})^2$, $(\sigma_0^{\text{end}})^2$, initial temperature $\tau^{(0)}$, t_i, t_f.

2: **Initialize:** $t = 1$, $T = \lceil En/m \rceil$, a stochastic gradient MCMC (SGMCMC) optimizer (Ma et al., 2015).

3: **Annealing the Prior:** Initialize $\boldsymbol{\theta}^{(t)}$ at $\boldsymbol{\theta}^{(0)}$, and simulate from a sequence of distributions $\pi(\boldsymbol{\theta}^{(t)}|D_n, \tau^{(t)}, \eta^{(t)}, (\sigma_0^{(t)})^2) \propto e^{nL(\boldsymbol{\theta}^{(t)}, D_n)/\tau^{(t)}} \pi_t^{\eta^{(t)}/\tau^{(t)}}(\boldsymbol{\theta}^{(t)})$ for $t = 1, 2, \ldots, t_i, \ldots, t_f, \ldots,$ T, where $0 < \eta^{(1)} \leqslant \eta^{(2)} \leqslant \cdots \leqslant \eta^{(t_i)} = \eta^{(t_i+1)} = \cdots = \eta^{(T)} = 1$, $\tau^{(0)} = \tau^{(1)} = \cdots = \tau^{(t_f)} \geqslant \cdots \geqslant \tau^{(T)}$, and $\pi_t = \lambda N(0, \sigma_1^2) + (1-\lambda) N(0, (\sigma_0^{(t)})^2)$, and $\sigma_0^{\text{init}} = \sigma_0^{(1)} \geqslant \sigma_0^{(2)} \geqslant \cdots \geqslant \sigma_0^{(t_f)} = \sigma_0^{(t_f+1)} = \cdots = \sigma_0^{(T)} = \sigma_0^{\text{end}}$. Denote the resulting model by $\boldsymbol{\theta}^{(t_f)}$.

4: **Structure Sparsification:** For each model parameter $i \in \{1, 2, \ldots, d\}$, set $\theta_i^{(t_f)} = 1$, if $|\theta_i^{(t_f)}| > \frac{\sqrt{2}\sigma_0\sigma_1}{\sqrt{\sigma_1^2 - \sigma_0^2}} \sqrt{\log\left(\frac{1-\lambda}{\lambda}\frac{\sigma_1}{\sigma_0}\right)}$ and 0 otherwise, and where $\sigma_0 = \sigma_0^{\text{end}}$.

5: **Input:** training dataset D_n, sparsified model $\boldsymbol{\theta}^{(t_f)}$, number of refining epochs E_r, mini-batch size m_r.

6: **Initialize:** $t = 1$, $T_r = \lceil E_r n/m_r \rceil$, an optimizer.

7: **Nonzero-weights Refining:** Refine the nonzero weights of the sparse model $\boldsymbol{\theta}^{(t_f)}$ by minimizing $L(\boldsymbol{\beta}^{(t_f)}, D_n)$.

In practice, for model pruning, one can utilize a standard optimizer such as Adam or AdamW, rather than employing stochastic gradient MCMC optimizers. A linear scheduler is implemented for $\eta^{(t)}$, $\tau^{(t)}$, and $\sigma_0^{(t)}$ (Sun et al., 2021; Zhang et al., 2023).

$$\sigma_0^{(t)}, \eta^{(t)}, \tau^{(t)} = \begin{cases} \sigma_0^{\text{init}}, \dfrac{t}{t_i}, \tau^{(0)} & t < t_i \\ \sigma_0^{\text{end}} + (\sigma_0^{\text{init}} - \sigma_0^{\text{end}}) \left(1 - \frac{t-t_i}{t_f-t_i}\right), 1, \tau^{(0)} & t_i \leqslant t \leqslant t_f \\ \sigma_0^{\text{end}}, 1, \dfrac{\tau^{(0)}}{t-t_f} & t_f < t \leqslant T \end{cases} \tag{S2.1}$$

S3 Experimental Information

In this section, we provide detailed information about our experiments, all of which were conducted on A100-80GB GPUs. For all experiments involving MGPP, we set λ to $1e-7$, and select the σ_0^2 from $\{1e-9, 1e-10\}$ and the σ_1^2 from $\{0.1, 0.05\}$. We choose the learning rates from the set $\{1e-4, 9e-5, 8e-5, 7e-5, 5e-5, 2e-5, 1e-5\}$ and select batch sizes from $\{8, 16, 32, 64\}$. Each experiment adheres to the same number of training epochs as described in (Li et al., 2023). For the hyperparameters of the cubic sparsity scheduler, we maintain the same Δt value as in (Li et al., 2023). Although we largely adhere to their t_i and t_f values, we make necessary adjustments based on our chosen batch size. We use the AdamW optimizer for all experiments.

S3.1 Natural Language Understanding

Table S1 provides the dataset statistics of the GLUE benchmark (Wang et al., 2018), while Table S2 details the training hyperparameters for DeBERTaV3$_{\text{base}}$, and Table S3 presents the training hyperparameters for BERT$_{\text{base}}$.

Table S1: Summary of the GLUE benchmark.

**Corpus**	Task	#Train	#Dev	#Test	#Label	Metrics
			Single-Sentence Classification (GLUE)			
CoLA	Acceptability	8.5k	1k	1k	2	Matthews corr (Mcc)
SST	Sentiment	67k	872	1.8k	2	Accuracy (Acc)
			Pairwise Text Classification (GLUE)			
MNLI	NLI	393k	20k	20k	3	Accuracy (Acc)
RTE	NLI	2.5k	276	3k	2	Accuracy (Acc)
QQP	Paraphrase	364k	40k	391k	2	Accuracy/F1 (Acc/F1)
MRPC	Paraphrase	3.7k	408	1.7k	2	Accuracy/F1 (Acc/F1)
QNLI	QA/NLI	108k	5.7k	5.7k	2	Accuracy (Acc)
			Text Similarity (GLUE)			
STS-B	Similarity	7k	1.5k	1.4k	1	Pearson/Spearman corr (P/S corr)

Table S2: Hyperparameter setup for MGPP on the GLUE benchmark to prune DeBERTaV3$_{\text{base}}$.

**Sparsity**	**Hyperparameter**	**MNLI**	**RTE**	**QNLI**	**MRPC**	**QQP**	**SST-2**	**CoLA**	**STS-B**
—	#epochs	8	20	10	10	10	6	5	5
	Batch size	32	16	64	16	32	32	32	16
	Δt	10	10	10	10	10	10	10	10
	t_i	5500	1000	1500	750	10000	2000	1500	1000
	t_f	75500	2500	11500	2300	85000	8000	3500	3500
	σ_0^2	1e-10	1e-10	1e-10	1e-9	1e-10	1e-10	1e-9	1e-9
	σ_1^2	0.05	0.1	0.1	0.1	0.1	0.1	0.1	0.1
80%	Learning rate	5e-5	1e-4	8e-5	9e-5	1e-4	7e-5	1e-4	1e-4
85%	Learning rate	5e-5	1e-4	8e-5	1e-4	1e-4	8e-5	1e-4	1e-4
90%	Learning rate	8e-5	1e-4	8e-5	1e-4	1e-4	1e-4	1e-4	1e-4

S3.2 Question Answering

Table S4 provides details of the training hyperparameters

Table S3: Hyperparameter setup for MGPP on the GLUE benchmark to prune $\text{BERT}_{\text{base}}$.

**Hyperparameter**	**MNLI**	**QQP**	**QNLI**	**SQuAD**	**SST-2**
epochs	8	10	10	10	6
Batch size	32	32	32	16	32
Δt	100	100	100	100	10
t_i	1 epoch	2 epoch	2 epoch	2 epoch	1000 iterations
σ_0^2	1e-10	1e-10	1e-9	1e-10	1e-9
σ_1^2	0.05	0.05	0.05	0.05	0.1
Learning rate	Linearly decay from 5e-5 to 5e-6				
λ	1×10^{-7}				

Table S4: Hyperparameter setup for MGPP on the SQuAD-v1.1 dataset to prune DeBERTaV3-base.

**Sparsity**	#epochs	Batch size	Learning rate	Δt	t_i	t_f	σ_0^2	σ_1^2
50%	10	16	5e-5	10	10000	35000	1e-10	0.05
60%	10	16	5e-5	10	10000	35000	1e-10	0.05
70%	10	16	5e-5	10	10000	35000	1e-10	0.05
80%	10	16	5e-5	10	10000	35000	1e-10	0.05
90%	10	16	5e-5	10	5000	40000	1e-10	0.05
95%	10	16	5e-5	10	5000	40000	1e-10	0.05

S3.3 Natural Language Generation

Table S5 provides details of the training hyperparameters

Table S5: Hyperparameter setup for MGPP on XSum/CNN_DailyMail datasets to prune BART-large.

Sparsity	Hyperparameter	XSum	CNN_DailyMail
70%, 60%, 50%	#epochs	12	12
	Batch size	32	32
	t_i	20000	20000
	t_f	60000	90000
	Δt	100	100
	σ_0^2	1e-10	1e-10
	σ_1^2	0.1	0.1
	Learning rate	2e-5	2e-5

S3.4 Upstream Pruning

Given the extensive size of the two datasets used in the upstream pruning process, pruning was carried out on 4 V100-32GB GPUs, with each epoch requiring approximately one day to complete. Due to computational resource constraints, we adopted the hyperparameters used for the MNLI dataset from our downstream pruning experiments, with adjustments including lowering σ_1^2 to 0.01 and setting the pruning frequency to every 200 iterations. For other relevant hyperparameters, we adhered to those utilized by oBERT.

- Batch size: 256.
- Learning rate: linear decay from 5e-5 to 5e-6.
- Maximum sequence length: 512.
- Number of epochs for pruning: 3.

For the sparse fine-tuning stage, we use the same hyperparameters across all datasets.

- Batch size: 32.
- Learning rate: linear decay from 2e-5 to 0.
- Maximum sequence length: 512.
- Number of epochs: 8.

S3.5 Ablation Study

For the L_2 ablation variant, we set the weight decay coefficient to $1e-2$, chosen from the set $\{0.1, 1e-2, 1e-3, 1e-4, 1e-5\}$. Additional hyperparameters are detailed in Table S6.

For the PA ablation variant, we conducted an extensive search for the optimal hyperparameters. The specific hyperparameters are detailed in Table S7.

Table S6: Hyperparameter setup for L_2 on the GLUE benchmark to prune DeBERTaV3-base.

Sparsity	Hyperparameter	MNLI	MRPC	SST-2
80%, 85%, 90%	#epochs	8	10	6
	Batch size	32	16	32
	Δt	10	10	10
	t_i	5500	1000	1500
	t_f	75500	2500	11500
	Learning rate	5e-5	9e-5	5e-5

Table S7: Hyperparameter setup for PA on the GLUE benchmark to prune DeBERTaV3-base.

Sparsity	Hyperparameter	MNLI	MRPC	SST-2
—	E	7	9	5
	m	32	16	32
	E_r	1	1	1
	m_r	32	16	32
	$\tau^{(0)}$	1	1	1
	λ	1e-7	1e-7	1e-7
	σ_1^2	0.05	0.1	0.5
	learning rate	2e-5	5e-5	2e-5
80%	t_i	5000	1000	1500
	t_f	80000	3000	12000
	$(\sigma_0^{\text{init}})^2$	1e-4	1e-4	1e-4
	$(\sigma_0^{\text{end}})^2$	1e-5	1e-5	1e-5
85%	t_i	5000	1000	1500
	t_f	80000	2500	12000
	$(\sigma_0^{\text{init}})^2$	1.2e-4	1.2e-4	1.2e-4
	$(\sigma_0^{\text{end}})^2$	2e-5	2e-5	2e-5
90%	t_i	5000	1000	1500
	t_f	80000	3000	12000
	$(\sigma_0^{\text{init}})^2$	1.4e-4	1.4e-4	1.4e-4
	$(\sigma_0^{\text{end}})^2$	3e-5	3e-5	3e-5

References

Li Y, Yu Y, Zhang Q, Liang C, He P, Chen W, et al. (2023). Losparse: Structured compression of large language models based on low-rank and sparse approximation. *arXiv preprint arXiv:2306.11222*.

Ma Y, Chen T, Fox EB (2015). A complete recipe for stochastic gradient MCMC. In: *Advances in*

Neural Information Processing Systems 28: Annual Conference on Neural Information Processing Systems 2015, December 7-12, 2015, Montreal, Quebec, Canada (C Cortes, ND Lawrence, DD Lee, M Sugiyama, R Garnett, eds.), 2917–2925.

Sun Y, Xiong W, Liang F (2021). Sparse deep learning: A new framework immune to local traps and miscalibration. In: *Advances in Neural Information Processing Systems 34: Annual Conference on Neural Information Processing Systems 2021, NeurIPS 2021, December 6-14, 2021, virtual* (M Ranzato, A Beygelzimer, YN Dauphin, P Liang, JW Vaughan, eds.), 22301–22312.

Wang A, Singh A, Michael J, Hill F, Levy O, Bowman SR (2018). Glue: A multi-task benchmark and analysis platform for natural language understanding. *arXiv preprint arXiv:1804.07461*.

Zhang M, Sun Y, Liang F (2023). Sparse deep learning for time series data: Theory and applications. In: *Advances in Neural Information Processing Systems 36: Annual Conference on Neural Information Processing Systems 2023, NeurIPS 2023, New Orleans, LA, USA, December 10 - 16, 2023* (A Oh, T Naumann, A Globerson, K Saenko, M Hardt, S Levine, eds.).
